# Molecular characterization of *Mycobacterium bovis* infection in cattle and buffalo in Amazon Region, Brazil

**DOI:** 10.1002/vms3.203

**Published:** 2019-09-30

**Authors:** Paulo A. M. Carneiro, Taynara. N. Pasquatti, Haruo Takatani, Martin J. Zumárraga, Maria. J. Marfil, Christian Barnard, Scott D. Fitzgerald, Robert B. Abramovitch, Flábio R. Araujo, John B. Kaneene

**Affiliations:** ^1^ Center for Comparative Epidemiology College of Veterinary Medicine Michigan State University East Lansing MI USA; ^2^ Amazonas State Federal Institute Manaus AM Brazil; ^3^ Catholic University Dom Bosco Campo Grande MS Brazil; ^4^ Agencia de Defesa Agropecuaria do Amazonas Manaus AM Brazil; ^5^ Instituto Nacional de Tecnología Agropecuaria (INTA) Instituto de Biotecnología Buenos Aires Argentina; ^6^ Veterinary Diagnostic Laboratory Michigan State University Lansing MI USA; ^7^ Department of Microbiology and Molecular Genetics Michigan State University East Lansing MI USA; ^8^ Centro Nacional de Pesquisa de Gado de Corte Campo Grande MS Brazil

**Keywords:** bovine tuberculosis, cattle and buffalo, genotypes, *Mycobacterium bovis*, spoligotyping

## Abstract

The aim of this study was to characterize *Mycobacterium bovis* from cattle and buffalo tissue samples, from two Brazilian states, and to analyse their genetic diversity by spoligotyping. Tissue samples from tuberculosis suspect animals, 57 in Amazonas State (12 cattle and 45 buffaloes) and six from Pará State (5 cattle and one buffalo) from slaughterhouses under State Veterinary Inspection, were isolated in culture medium Stonebrink. The positive cultures were confirmed by PCR and analysed by the spoligotyping technique and the patterns (spoligotypes) were identified and compared at the *Mycobacterium bovis* Spoligotype Database (http://www.mbovis.org/). There was bacterial growth in 44 (69.8%) of the tissues of the 63 animals, of which PCR for region of differentiation 4 identified 35/44 (79.5%) as *Mycobacterium bovis*. Six different spoligotypes were identified among the 35 *Mycobacterium bovis* isolates, of which SB0295, SB1869, SB0121 and SB1800 had already been described in Brazil, and SB0822 and SB1608 had not been described. The most frequent spoligotype in this study (SB0822) had already been described in buffaloes in Colombia, a neighbouring country of Amazonas state. The other identified spoligotypes were also described in other South American countries, such as Argentina and Venezuela, and described in the Brazilian states of Rio Grande do Sul, Santa Catarina, São Paulo, Minas Gerais, Mato Grosso do Sul, Mato Grosso and Goiás, indicating an active movement of *Mycobacterium bovis* strains within Brazil.

## Graphical abstract

The aims of the study are to determine the genetic diversity of *Mycobacterium bovis* isolates from cattle and buffalos in the states of Amazonas and Para in Brazil, using spoligotyping, and to compare the spoligotypes to those reported in other South American countries. Six molecularly different spoligotypes were identified: SB0295, SB1869, SB0121, SB1800, SB0822 and SB1608. Two of the six (SB0822 and SB1608) had not been reported in Brazil. Spoligotype SB0822 had been reported in a neighbouring country (Columbia), suggesting a possible movement of animals between the countries.

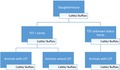

## 1 INTRODUCTION

Bovine tuberculosis (BTB) is a chronic, infectious disease caused by *Mycobacterium bovis (M. bovis)*, a member of a group called the *Mycobacterium tuberculosis* Complex (MTC), which includes tuberculosis‐causing mycobacteria such as *M. tuberculosis*, *M. canettii*, *M. africanum*, *M. pinnipedii*, *M. microti*, *M. caprae*, *M. bovis*, *M. suricattae*, *M. mung*i and *M. orygis* (Dawson et al., 2012; Rodrigues, 2017).

Bovine tuberculosis predominantly affects cattle and buffaloes, but may occasionally infect other mammalian species, including humans (Etchechoury et al., 2010; Silva et al., 2018). It may spread through direct contact with infected animals, causing the spread of disease among herds or herds to wild animals and vice versa (Corner, Murphy, & Gormley, 2011; Reis, 2015), or being transmitted through indirect contact with contaminated equipment, water and food (Palmer, 2013; Smith, Tauer, Schukken, Lu, & Grohn, 2013).

Globally recognized, BTB persists in both developed and developing countries (Broughan et al., 2013; Michel, Müller, & Helden, 2010). In Brazil, the Ministry of Livestock and Food Supply was established in 2001 and modified in 2017 the National Program for the Control and Eradication of Brucellosis and Animal Tuberculosis, to reduce the prevalence and incidence of BTB (Carneiro & Kaneene, 2018). The regulation determines the slaughter of all bovines and buffaloes that present a positive reaction to the tuberculin test (ante mortem diagnosis) and as gold standard, isolation in culture medium for identification and confirmation of *M. bovis* infection (post‐diagnosis—mortem) (Brasil, 2017).

Molecular techniques are increasingly used to support conventional methods, both for the identification and confirmation of *M. bovis* strains, and for molecular epidemiology. Molecular genotyping by spoligotyping is a technique developed by Kamerbeek et al. (1997) which discriminates genotypes of *M. bovis* by amplification of the polymorphic DR (Direct Repeats) chromosomal locus in the MTC which contains DR sequences interspersed with variable spacer sequences, followed by a reversed line blot hybridization (RLBH). The presence or absence of the spacers is identified. Spoligotyping, by discriminating *M. bovis* genotypes, through RLBH patterns, may aid in BTB control programs, providing epidemiological data among isolates. Therefore, this study was designed to determine the spoligotypes of *M. bovis* isolates from cattle and buffalos in the Amazon region of Brazil.

## 2 METHODS

### 2.1 Sample collection

**Figure 1:**
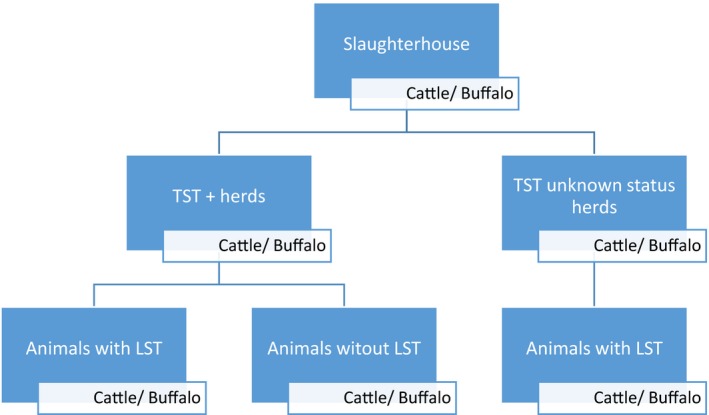
Diagram of the sampling process

From July 2016 to February 2017, a total of 922 animals were inspected (635 cattle and 287 buffaloes), from those 63 samples of cattle (*n* = 17) and buffalo (*n* = 46) tissues were obtained. From the herds with report of TST reactive animal samples of all animals sent to the slaughterhouses, with or without lesions suggestive of tuberculosis (LST), were collected. From herds with unknown TST status samples were collected only from animals with LST (Figure 1). Three abattoirs were selected based on logistics and willing to participate. The inspection of the animals was performed by trained officials of Amazonas State Veterinary Inspection Service, LST were defined as granulomas small, spherical, tan and firm nodules usually with a mineralized core. The same criteria for detection of lesions were used for cattle and buffaloes.

Two samples per animal were collected (one from the suspected tissue and one from the retropharyngeal lymph node) and the unit of analysis was the animal. For the analysis, herd was considered infected when it presented at least one animal confirmed positive by the PCR analysis. The animals were slaughtered for commercial purposes. Thus, there was no animal sacrifice due to this study.

Herds from 10 municipalities of Amazonas and four municipalities in Para were involved (Table 1). The median age group of inspected animals in both species was from 25 to 36 months old, the mean herd size was 142 for cattle and 84 for buffaloes. Unfortunately, we have only data available from Amazonas State, the municipalities on the study have 3,818 cattle herds and 1,315 buffalo herds.

**Table 1:** *M. bovis* culture, PCR, and prevalence by species

Species	No. of Animals Inspected	No. of animals from which the samples were collected^a^	No. of animals with Legion Suggestive of Tuberculosis (LST)	Culture +	PCR +	Sample Prevalence
Cattle	635	17	13	9	8	1.26%
Buffalo	287	46	33	27	27	9.41%
TOTAL	922	63	46	36	35	3.8%

Abbreviation: LST, lesions suggestive of tuberculosis.

a 13 cattle and 33 buffaloes had LST. Two samples per animal were collected. One cattle and 9 buffaloes without LST were PCR +.

The samples were shipped in a sterile plastic packages containing the Animal Transit Guide number, which has info about animal species, sex and age but no information about tissue and race. The samples were transported under refrigeration in a thermic container with artificial ice to the Animal Immunology Laboratory of Embrapa Beef‐Cattle, located in Campo Grande, MS, for further analysis.

### 2.2 Preparation and culture of samples

Lesions suggestive of tuberculosis (10–25 mg) were macerated in 2 ml tubes containing ceramic beads (MagNA Lyser green beads) and 1 ml of sterile water in a MagNA Lyser Instrument (Roche) for three cycles of 30 s at 6.000 rpm. Later, 1 ml of 1N NaOH was added, and the tube was incubated at 37°C for 15 min. The tube was centrifuged at 3,000 rpm for 15 min, and the supernatant was discarded. The decontamination by Petroff method was performed. Briefly, the pellet was suspended in 1 ml of sterile distilled water and 100 µl of 0.2% phenol red solution was added. After that, 50–100 µl of 1% HCl was added until change of colour was visualized—from pink to amber yellow. The pH was adjusted to 7.0 with neutralizing solution and 300 µl of the material was inoculated in duplicate into the Stonebrink medium (de Kantor et al., 1998). The Stonebrink medium has the same composition as Lowenstein–Jensen, except that glycerol is replaced by 0.5% sodium pyruvate, further incubated at 37°C, and evaluated weekly for 90 days to verify bacterial growth. One medium per sample was used. The colonies with characteristics suggestive of *M. bovis* were submitted to DNA extraction.

### 2.3 DNA extraction

The bacterial colonies were washed with 500 μl of Tris‐EDTA buffer in microtubes and inactivated in a dry bath for 1 hr at 87°C, with subsequent centrifugation at 14,000 rpm for 2 min. The pellet that formed was discarded and the supernatant containing the mycobacterial DNA was transferred to new microtubes and stored at −20°C for subsequent analysis.

This DNA extraction method has been reported by our laboratory and others (Lamine‐Khemiri et al., 2013; Shimizu et al., 2014; Yahyaoui‐Azami et al., 2017; Zumárraga et al., 2013).

### 2.4 Microorganism identification by PCR

The mycobacterial DNA samples were submitted to standard PCR according to Sales et al. (2014), using primers Mb.400.F (5′AACGCGACGACCTCATATTC3′) and Mb.400.R (5′AAGGCGAACAGATTCAGCAT3′), which amplify a 400 base pair DNA fragment flanking the region of differentiation 4, specific to *M. bovis* (Ramos et al., 2014). The PCR products were stained with Gel Red and submitted to 1% agarose gel electrophoresis in 1× TAE buffer and visualized in a PhotoDocumentor under ultraviolet light.

### 2.5 Spoligotyping

**Figure 2:**
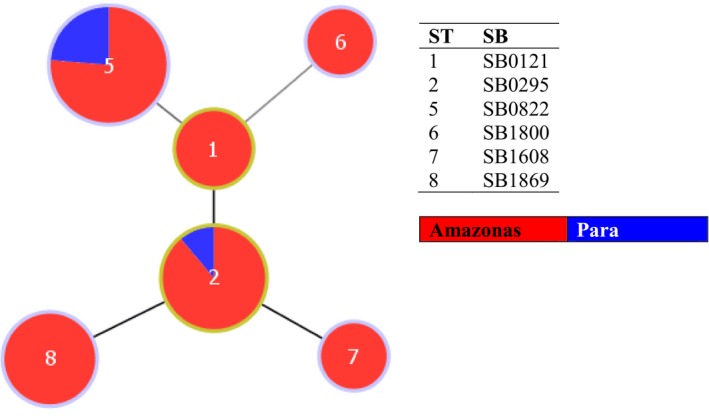
e‐Burst of the 35 M*. bovis* isolates from the Amazon region of Brazil

The spoligotyping was performed on *M. bovis* isolates, following the instructions of Kamerbeek et al. (1997). Hybridization of the PCR product was performed on a spoligotyping membrane with oligonucleotides of spacer sequences, using a miniblotter according to the manufacturer's instructions (MapMyGenome). The membrane was incubated with streptavidin‐peroxidase and the spacers were detected by ECL chemiluminescence (Pierce ECL Western Blotting Substrate, Thermo Fisher Scientific), followed by exposure of an X‐ray film to the membrane. The patterns visualized in the X‐ray film were compared to those contained in the database on the Mbovis.org website (http://www.mbovis.org/) of Complutense University of Madrid, Spain. To analyse and visualize the hypothetical relationship of genetic patterns of the strains, we have applied an eBURST algorithm using the PhyloViz free software (Figure 2) (Francisco et al., 2012).

## 3 RESULTS AND DISCUSSION

The study was based on a convenience sampling of adult animals sent to three major slaughterhouses in Amazonas State, those slaughterhouses represent more than 70% of all regional cattle and buffaloes slaughtered on the region. All herds for the two species, but one, on this study came from the Geographical region known as Low Amazon River which comprehends the East region of Amazonas State and West Region of Para State. The sampling process was performed throughout the rain and dry season which would reduce any potential bias due to the seasonality during the sampling. Moreover, during the dry season, cattle and buffalo herds are concentrated on the floodplains grassland where they have access to high‐quality pasture and naturally gain weight and improve the immune defences what could represent a counterbalance to the overexposure to animals from diverse herds with unknown BTB status. On the other hand, during the Rainy season, although less exposed to BTB transmission between herds, the probability of transmission within the herd can increase due to less favourable offer of pasture and proximity of animals during dairy offer of food supplementation.

The regional herds, cattle and buffaloes, are mainly managed in extensive system characterized by farms with low technological level and productivity and few managed in semi‐intensive systems characterized by farms with good technological level and productivity. In common, the two systems have the influence of the river floods. During the water floods (November to June), herds remain at the mainland areas, and during the dry season (July to mid‐November) weaned calves, steers, heifers and dry cows are transported to floodplain's grassland for beef or recovery purpose which represents an additional challenge regarding the infection by *M. bovis* because the floodplain's grassland are shared between herds from several owners with no guarantee of sanitary control. Apuí is the only municipality on this study out of the influence of the Amazon River, with herds in a semi‐intensive system managed only in grasslands not subject to flooding season. Although, not including animals bellow two years old, the sample can be considered representative to determine the status of BTB on regional herds, since the median age of the sample for both species in the study are similar the median age of the respective reference population (25–36 months) and though BTB can occurs in young cattle and buffalo its commonly affect adult animals.

All the herds in the sampling areas are from open herds with frequent introduction of animals from other herds and regions. All, but one, share pasture during the low season of Amazon River and/or those factors can represent a major factor for the BTB dissemination. Modern modelling studies in England reveal that movement of infected animals was responsible for 84% of newly infected farms (Brooks‐Pollock, Roberts, & Keeling, 2014). Disease control measures, basically, are reduced to the vaccination against foot and mouth disease twice a year, and control measures against tuberculosis, such as diagnostic and elimination of positive animals, are not adopted regularly. Some herds of the study presented control of brucellosis by vaccination of the heifers with B19 vaccine.

In all, 63 samples from 17 cattle and 46 buffaloes, from 25 herds, were isolated in Stonebrink culture medium. In total, 14 showed no growth, 13 contaminated cultures were discarded due to presenting growing compatible with environmental contamination and 36 show growth of colony compatible with Mycobacterium. In addition, in Amazonas State, 7 from 10 municipalities presented positive results and in Para 2 from 4 municipalities presented positive results, showing that the disease is widespread in the region.

The study was based on a convenience sampling performed during the routine inspection service at the slaughterhouses. Samples were collected from all animal that came from herds with TST reactive status. However, due to logistic and financial reasons, animals from unknown Tb status herds, samples were collected only from animas that had lesions. No information about the BTB prevalence on the reactive herds were provided. The authors recognize the situation might be a limitation of the study since the prevalence can be artificially increased if the samples without LST from TST reactive herd presented a significant difference of positive samples; however, in our analysis, this situation was not observed.

PCR results confirmed a total of 35 animals (3.8%) positive for *M. bovis* in 4 isolates of cattle and 27 buffaloes in the state of Amazonas and 4 cattle from Para State, which means a prevalence within species of 1.26% in cattle and 9.41% in buffaloes (Table 2). The result seems to confirm the regional belief that *M. bovis* has higher occurrence in buffaloes over cattle in the area. The municipalities with highest prevalence were Autazes (17.86%), Urucara (12.06%), Itacoatiara (5.88%) and Apui (4.2%) in Amazonas State and Prainha (3%) in Para.

**Table 2:** Distribution of the 35 *M. bovis* isolates from the Amazon region, according to place of origin, number of animals inspected, species, presence of lesion, and spoligotype found

Municipality/state	Animals inspected	Species	Lesion	Spoligotype (*n*)
Alenquer/PA	150	Cattle	+	SB0822 (1)
Praínha/PA	100	Cattle	+	SB0822 (3)
Apuí/AM	162	Cattle	+	SB1800 (1)
Autazes/AM	184	Buffalo	+	SB1869 (1)
		Buffalo	−	SB1869 (1)
		Buffalo	+	SB0121 (1)
Autazes (NCD)/AM	213	Buffalo	+	SB1869 (1)
		Buffalo	−	SB1869 (1)
		Buffalo	+	SB0822 (2)
		Buffalo	−	SB0822 (2)
		Buffalo	+	SB0295 (2)
		Buffalo	−	SB0295 (3)
		Buffalo	+	SB0121(1)
Careiro da Várzea/AM	98	Cattle	−	SB0822 (1)
Itacoatiara/AM	17	Buffalo	+	SB1869 (1)
Manacapuru/AM	298	Cattle	+	SB0822 (2)
Parintins/AM	50	Buffalo	+	SB1608 (1)
		Buffalo	−	SB0295 (3)
Urucará/AM	58	Buffalo	−	SB0295 (1)
		Buffalo	+	SB0822 (5)
		Buffalo	−	SB0822 (1)

Abbreviation: Autazes (NCD)/AM, Autazes (Novo Céu District)/Amazonas.

The prevalence in buffaloes seems to agree with previous studies in the region (Barbosa et al., 2013; Mota et al., 2002); however, in the first study, out of 266 skin test reagent animals, only 14 were sacrificed for microbiological analysis and in our second study, only TST test was performed and all TST reagent animals were tested for microbiological and molecular diagnosis of *M. bovis*. In our study, the higher prevalence in buffalos might be explained by three factors: an environmental factor—in this study, buffalo herds had less herd health than cattle herds; a behavioural factor—buffaloes under pasture have a high tendency to stay closer to each other than cattle which favours the transmission of the *M. bovis*, or a genetic factor, buffalo can be more susceptive to specific *M. bovis* strain.

**Figure 3:**
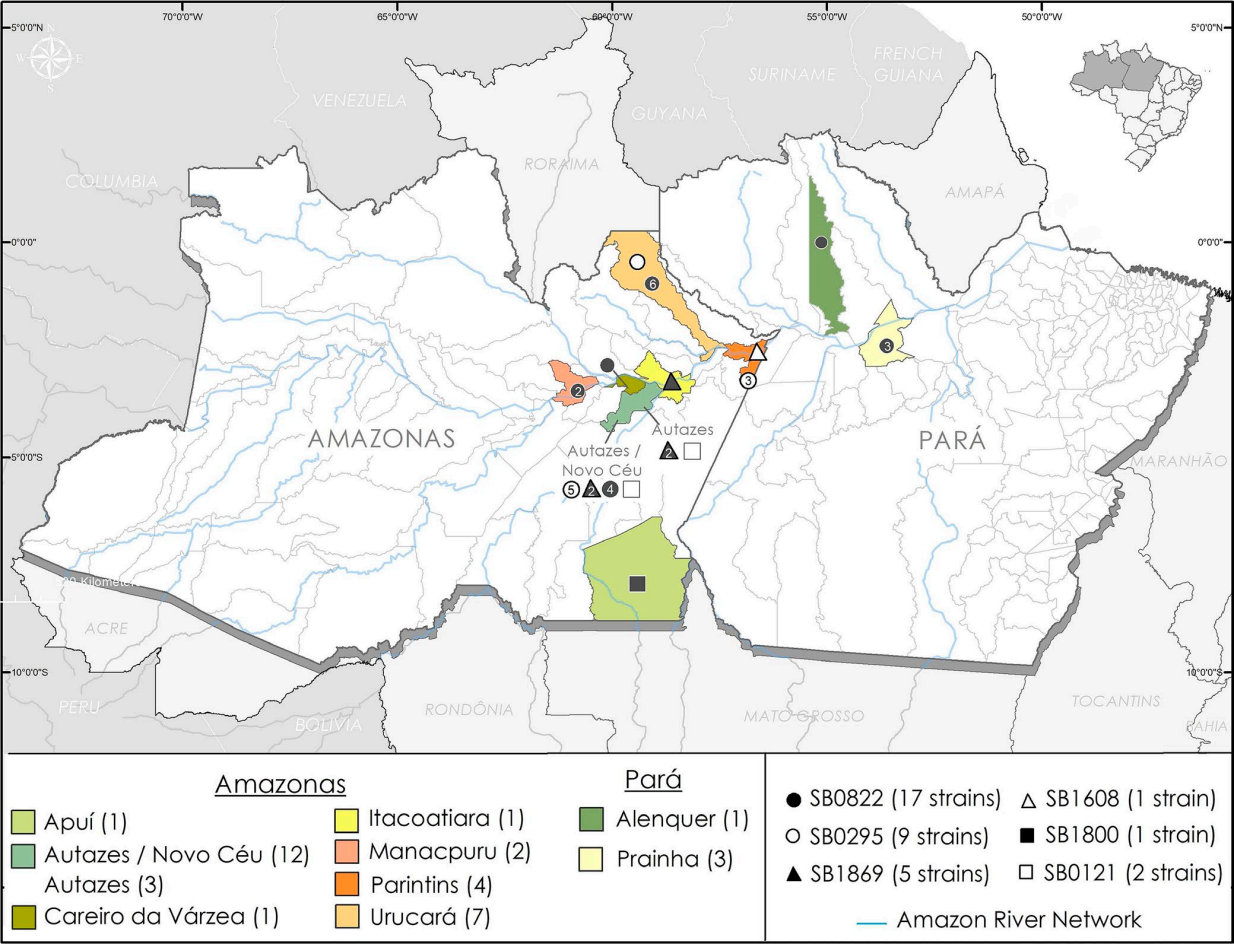
Geographical origin and spoligotypes of the *M. bovis* isolates from municipalities of Amazon region, Brazil

The spoligotyping was performed on 35 *M. bovis* isolates and six types of spoligotypes were identified: SB0822, SB0295, SB1869, SB0121, SB1800 and SB1608 (Table 1). The geographical distribution of the strains is shown in Figure 3. The municipality of Autazes presented the largest variety of strains with four spoligotypes, followed by Parintins and Urucará with two spoligotypes each. The other municipalities presented only one spoligotype each, Manacapuru (SB0822), Itacoatiara (SB1869), Careiro da Várzea (SB0822), and Apuí (SB1800) in Amazonas state, and the municipalities of Alenquer and Praínha in Pará state presented the same spoligotype SB0822. The district of Novo Ceu in the municipality of Autazes presented the largest variety of isolates, with four different spoligotypes in the studied animals, SB0295 (*n* = 5), SB0822 (*n* = 4), SB1869 (*n* = 2) and SB0121 (*n* = 1), highlighting a condition to facilitate the dissemination of distinct genotypes in the region. The geographical location at the border of two municipalities and with great availability of pasture in flood lands which attracts in transit herds that mingle in the area can be the reason for the prevalence rate and certainly should be particularly observed by the regional Bovine TB control programme. The municipalities of Alenquer, Praínha, Careiro da Várzea, Urucará, and Manacapuru and Itacoatiara, located at the border of the Amazon River, presented shared spoligotypes reflecting the intense transit of animals through the river. Moreover, the municipality of Apuí, located at the Southwest region of Amazonas state and not linked to the Amazon basin, presented a unique profile suggesting that other factors might drive the *M. bovis* distribution in that area.

The spoligotype SB0822, although considered unusual, was the most commonly observed in this study. This spoligotype was present in the Amazonas municipalities of Careiro da Várzea in one cattle, Manacapuru in two cattle, Urucará in six buffaloes and Autazes/ Novo Céu district in four buffaloes. In the state of Pará, one cattle was observed in the municipality of Alenquer and three in the municipality of Praínha, all with LST. This spoligotype had not been described in Brazil, but has been previously described in France (http://www.mbovis.org), cattle in Spain (Romero et al., 2008) and Portugal (Matos et al., 2010), and buffalo in Colombia (Jojoa‐Jojoa, Maira, Francisco, Puerto‐Castro, & Guerrero‐Guerrero, 2016), suggesting an active transmission of the strain between the animals of the Amazon region and the neighbouring country; however, there are neither roads nor known transit of cattle or buffaloes from Colombia to the region of the lower Amazon river. This link between Colombia and the area of study needs to be further explored.

Other spoligotypes considered unusual were observed in this study, as SB1869, which had already been described in São Paulo by Rocha (Rocha, 2013), was identified in five buffaloes (14.3%) in the state of Amazonas, one in Itacoatiara, two in Autazes and two in Autazes/Novo Ceu district (Table 1). Also considered unusual, SB1608 and SB1800 spoligotypes were isolated in this study, the spoligotype SB1608 isolated in buffalo in the municipality of Parintins had not previously been described in Brazil and was described in wild animals in Portugal (Carvalho et al., 2016), SB1800 was described in Brazil (http://www.mbovis.org) and identified in cattle in Apuí‐AM (Table 1).

SB0295 was the second most frequent spoligotype observed, with nine buffaloes in Amazonas, and was most frequently found in animals without LST (7/9). This spoligotype is considered the second most frequent in Brazil (Zumárraga et al., 2013), described in the state of Paraíba (Higino et al., 2011), Bahia (Filho et al., 2014), Mato Grosso, Goiás (Carvalho et al., 2016), Mato Grosso do Sul, Santa Catarina (Parreiras et al., 2012) and the state of São Paulo (Rocha, 2013), but not described or identified in the states of Amazonas and Pará. Outside of Brazil, it was described in buffaloes in Argentina (Zumárraga et al., 2013) and alpacas (*Lama pacos*) (García‐Bocanegra et al., 2010), and wild boars (*Sus scrofa*) in Spain (García‐Bocanegra et al., 2012).

The spoligotype SB0121 is considered the most prevalent in several studies and the most frequent in Brazil (Zumárraga et al., 2013) and was observed in two (5.7%) buffaloes in the municipality of Autazes, as well as described in other studies, in the states of Bahia (Filho et al., 2014), Paraíba (Parreiras et al., 2012), Mato Grosso, Mato Grosso do Sul (Cazola et al., 2015), Goiás (Carvalho et al., 2016), Minas Gerais (Parreiras et al., 2012) and Rio Grande do Sul (Ramos et al., 2014). Outside of Brazil, SB0121 has been described in Colombia (Jojoa‐Jojoa et al., 2016), Argentina, Venezuela (Zumárraga et al., 2013), Mexico (Reyes et al., 2012), Portugal (Reis, 2015) and France (Hauer et al., 2015). In addition, the spoligotype SB0121 was identified as an agent of human tuberculosis in England (Stone, Brown, & Drobniewski, 2011) and the United States (Wilkins et al., 2008).

The SB0822 and SB0295 spoligotypes were together responsible for 74.3% of strains isolated in the Amazon region. SB1869, considered not very frequent, was the third most common spoligotype observed in this study, with 14.3% of the isolates in opposite to other studies describing SB0121 as the most prevalent, which comprised only 5.7% of the isolates in this study.

Considering that the evolution of the mycobacteria has occurred by successive loss of DNA, the founder spoligotypes would have more spacers than their descendants. The e‐BURST can be used with multilocus data to define groups or clonal complexes of related isolates derived from a common ancestor, the patterns of descent linking them together, and the ancestral genotype. The lack of the spacer N°37 from SB0121 would have evolved in the SB0295. On the other hand, the SB0295 could be considered a subgroup founder of spoligotypes SB1608 (lack of spacer N°15) and SB1869 (lack of spacers N°1 and 2). The spoligotype SB0121 could be the founder of SB0822 and SB1800; however, to explain their relationship, the presence of other spoligotypes not detected in this study should be considered (Figure 2). The spoligotyping was the option of choice for genotyping in this study, because after the growth of the isolates in culture, this technique was produced in a short period of time, demonstrating speed and ease. Offering data of diverse strains of *M. bovis* in broad scale confirmed the existing polymorphism between strains of the Amazon region.

In this study, it was possible to observe a high genetic diversity of *M. bovis* isolates in the Amazon region, including the detection of unusual spoligotypes in Brazil. It also detected a spoligotype found in Colombia, border country with the Amazon region, as well as identical genotypes in the two states of Pará and Amazonas. These facts suggest a possible dissemination of *M. bovis* genotypes by trade/transport of cattle between regions or perhaps a wildlife reservoir might be playing a role in the spatial distribution of *M. bovis* genotypes in the region.

## 4 CONCLUSIONS

- A high genetic diversity of *M. bovis* isolates were found in the Brazilian Amazon.
- The data corroborate with the previous information that buffaloes are more infected than cattle in the region.
- Genotype isolates in this study were reported in neighbouring countries suggesting the need for more studies to clarify the routes of transmission between regions.

## CONFLICT OF INTEREST

The authors declare no conflicts of interest.

## ETHICS STATEMENT

The authors confirm that the ethical policies of the journal, as noted on the journal's author guidelines page, have been adhered to and the appropriate ethical review committee approval has been received. The US National Research Council's guidelines for the Care and Use of Laboratory Animals were followed.
